# Mastoid vibration affects dynamic postural control during gait in healthy older adults

**DOI:** 10.1038/srep41547

**Published:** 2017-01-27

**Authors:** Jung Hung Chien, Mukul Mukherjee, Jenny Kent, Nicholas Stergiou

**Affiliations:** 1 Physical Therapy Education, University of Nebraska Medical Center, 42^nd^ and Emile, Omaha, NE 68198, USA; 2 Center for Research in Human Movement Variability, College of Education, University of Nebraska at Omaha, 6160 University Dr., Omaha, NE 68182-0860, USA; 3 College of Public Health, Department of Environmental, Agricultural & Occupational Health University of Nebraska Medical Center, 984355 Nebraska Medical Center, Omaha, NE 68198, USA.

## Abstract

Vestibular disorders are difficult to diagnose early due to the lack of a systematic assessment. Our previous work has developed a reliable experimental design and the result shows promising results that vestibular sensory input while walking could be affected through mastoid vibration (MV) and changes are in the direction of motion. In the present paper, we wanted to extend this work to older adults and investigate how manipulating sensory input through mastoid vibration (MV) could affect dynamic postural control during walking. Three levels of MV (none, unilateral, and bilateral) applied via vibrating elements placed on the mastoid processes were combined with the Locomotor Sensory Organization Test (LSOT) paradigm to challenge the visual and somatosensory systems. We hypothesized that the MV would affect sway variability during walking in older adults. Our results revealed that MV significantly not only increased the amount of sway variability but also decreased the temporal structure of sway variability only in anterior-posterior direction. Importantly, the bilateral MV stimulation generally produced larger effects than the unilateral. This is an important finding that confirmed our experimental design and the results produced could guide a more reliable screening of vestibular system deterioration.

Falls are a major focus of geriatric medicine because they are common among older adults, and often have serious consequences, including morbidity and disability^1^. Because falls often occur while walking, and poor gait performance is associated with falling, efforts are needed to address the increased gait unsteadiness in community-dwelling elderly fallers^1^. During the last thirty years considerable effort has been devoted to identifying sensitive measures of gait instability (e.g. gait speed, stride time variability)^2^,^3^. Less effort has been made towards identifying the mechanisms that could contribute to this gait instability. Specifically, it remains unclear how aging affects the contributions of the sensory systems that are involved in the control of gait^4^,^5^,^6^. Recently, we developed the Locomotor Sensory Organization Test (LSOT); an experimental paradigm, to study these contributions with more precision^7^,^8^. The LSOT allows manipulation of the visual and somatosensory inputs to study their effects on postural control during walking, paralleling the Sensory Organization Test (SOT) which is a widely used clinical test for examining such effects on standing posture^9^. The LSOT contains 6 conditions as following sequence: (1) normal walking, (2) walking with reduced vision, (3) walking with perturbed vision, (4) walking with perturbed somatosensation, (5) walking with reduced vision and perturbed somatosensation, and (6) walking with perturbed vision and perturbed somatosensation^7^.

Our previous work with the LSOT has shown that dynamic balance control during walking is affected by the systematic manipulation of multisensory inputs^7^,^8^. The amount of sway variability observed during walking reflects similar balance performance to standing posture^7^, indicating that similar feedback processes may be involved. However, the contribution of visual input is significantly higher during walking in comparison to standing^7^. Thus, we suggest that vision is the predominant sensory system in walking^7^. Our results also revealed that as sensory conflict increases, more rigid and regular sway patterns are found during standing, while the opposite is the case with walking, where more exploratory and adaptive movement patterns are observed^8^. However, these studies have only been performed with healthy young adults and thus the effect of aging on the responses to these sensory perturbations has not been investigated.

An additional unclear from these experiments was the involvement of any type of input from vestibular signals, as such contributions are not manipulated systematically with the LSOT (or the SOT). The contribution of the vestibular system is particularly important to consider in older adults. Previous work has found that the density of the labyrinthine hair cell receptors gradually decreases from as early as 30 years of age, followed by a steep decline in the number of vestibular receptor ganglion cells beginning around the ages of 55 to 60 years^10^. By the age of 70, only 60% of the hair and nerve cells of the vestibular system remain^11^. The deteriorated vestibular system produces impaired balance and dizziness. Particularly, it has been shown that older adults demonstrate significantly increased postural sway during standing and experience dizziness when visual and somatosensory systems are conflicted simultaneously^12^. A deteriorated vestibular system could result in self-orientation that is less reliable. It could also impair the ability to integrate sensory information reducing the capacity to compensate for discordant input^13^. Therefore, it is important to incorporate a manipulation of vestibular input to investigate this system’s contribution to walking performance especially when the focus is older adults. Recently, we have incorporated Mastoid Vibration (MV) to our LSOT experimental paradigm to address this issue.

In a previous study, we observed significant increases in measures of both the amount of sway variability and the temporal structure of sway variability in the anterior-posterior direction during walking on application of MV^14^. Bilateral MV produced larger effects than unilateral stimulation. Furthermore, for all conditions that involved visual and/or somatosensory manipulations, MV augmented the effect regardless of whether it was presented unilaterally or bilaterally. Again, this study was performed only with healthy young adults and thus the effect of aging has not been investigated^14^.

The augmentation of the LSOT with MV, that offers a vestibular challenge component, provides a comprehensive assessment of sensory integrity. The purpose of the present study was to utilize this combined paradigm to explore the contributions of the sensory systems to dynamic postural control of older adults during gait. Based on our previous work^7^,^8^ we hypothesized that the MV would significantly increase both the amount and temporal structure variability of postural sway during walking in older adults when any sensory system was manipulated. Moreover, while both visual and somatosensory systems were conflicted simultaneously in walking, we hypothesized that the effect of MV might be amplified in older adults when compared to our previous observations in young adults^14^. We also expect that bilaterally applied MV will produce larger effects than unilaterally applied MV.

## Results

### Normality Test

Shapiro-Wilk Test results were greater than 0.05 in all dependent variables indicating that the data were normally distributed (p = 0.706 for mean sway area, p = 0.202 for amount of sway variability, p = 0.054 for structure of sway variability in the AP direction, p = 0.091 for structure of sway variability in the ML direction).

### MV effects (Hypothesis #1)

We hypothesized that MV would significantly increase both the amount and temporal structure of variability of postural sway during walking.

### Mean sway area

There was no significant MV main effect on mean sway area (Table 1).

### Amount and structure of sway variability

**Figure 1:**
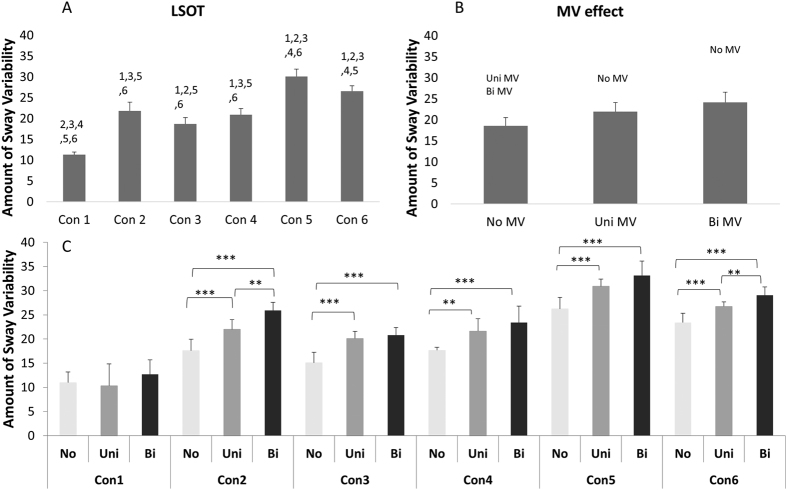
(**A**) Marginal means (averaging the three MV conditions) for the coefficient of variation of the six LSOT conditions. Error bars show standard deviations. The post hoc differences are indicated over the bars with the number of the condition with which differences were found. (**B**) Bar charts showing the marginal means (averaging the six LSOT condition) of the coefficient of variation of the three MV conditions. Error bars show standard deviations. The post hoc differences are indicated over the bars with the type of the condition with which differences were found. (**C**) Group means (cell means in terms of the two-way ANOVA) for all conditions with brackets over the bars to identify significant differences between conditions. **: <0.01; ***: <0.0001.

**Figure 2:**
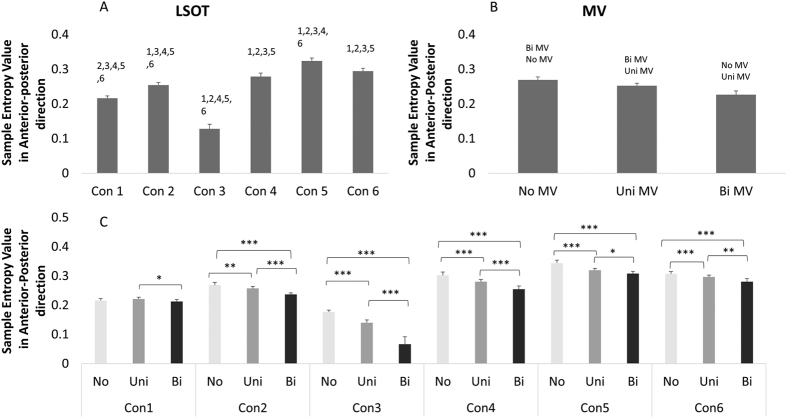
Marginal means (averaging the three MV conditions) for the Sample Entropy in the AP direction for the six LSOT conditions. Error bars are standard deviations. The post hoc differences are indicated over the bars with the number of the condition with which differences were found. (**B**) Marginal means (averaging the six LSOT condition) for the Sample Entropy in the AP direction for the three MV conditions. Error bars show standard deviations. The post hoc differences are indicated over the bars with the type of the condition with which differences were found. (**C**) Group means (cell means in terms of the two-way ANOVA) for all conditions. Brackets indicate significant differences between conditions. **: <0.01; ***: <0.0001.

**Figure 3:**
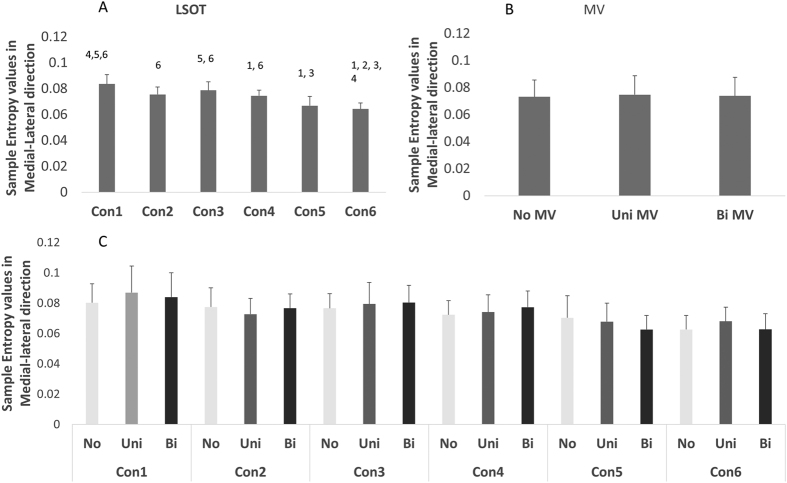
(**A**) Marginal means (averaging the three MV conditions) for the Sample Entropy in the ML direction for the six LSOT conditions. Error bars show standard deviations. The post hoc differences are indicated over the bars with the number of the condition with which differences were found. (**B**) Marginal means (averaging the six LSOT condition) for the Sample Entropy in the ML direction for the three MV conditions. Error bars show standard deviations. No significant main effect was found. (**C**) Group means (cell means in terms of the two-way ANOVA) for all conditions. No significant interaction was found.

A significant MV main effect was found in amount of sway variability (F = 162.39, p < 0.0001, Fig. 1B), in structure of sway variability in the anterior-posterior direction (F = 288.72, p < 0.0001, Fig. 2B) but not in structure of sway variability in the medial-lateral direction (Fig. 3B). The MV effect significantly increased the amount of postural control variability in older adults during gait. However, the MV effect decreased the structure of postural sway variability in anterior-posterior direction and didn’t affected those in medial-lateral direction (Figs 1B, 2B and 3B).

### LSOT condition effect (Hypothesis #2)

We hypothesized that the effect of MV might be larger in older adults while both visual and somatosensory systems were conflicted simultaneously in walking than while receiving other single-perturbed or no perturbed LSOT conditions. In addition, this aforementioned effect of MV might be amplified in older adults when compared to our previous observations in young adults while both visual and somatosensory systems were conflicted simultaneously.

### Mean sway area

A significant LSOT main effect (F = 5.68, *p* < 0.0001) was found (Table 1). The post hoc analysis revealed LSOT condition 5 had significantly smaller values than LSOT condition 1. In addition, the mean sway area was smaller in older adults (0.042 ± 0.005 m^2^) than in young adults^14^ (0.049 ± 0.008 m^2^) (Table 1).

### Amount and structure of sway variability

A significant LSOT main effect was found in amount of sway variability (F = 219.90, *p* < 0.0001, Fig. 1A), in structure of sway variability in anterior-posterior direction (F = 1632.99, *p* < 0.0001, Fig. 2A) and in medial-lateral direction (F = 21.87, *p* < 0.001, Fig. 3A). For amount of sway variability, post-hoc analysis revealed that the significantly larger group means were observed in condition 5, and 6 than in other conditions, whilst the smallest was found for condition 1. Similarly, for structure of sway variability in the anterior-posterior direction, post-hoc analysis revealed that significantly larger group means in condition 5 and 6 than other conditions. However, for structure of sway variability in the medial-lateral direction, group means were significantly lower in condition 5 and 6 than in condition 1 (Figs 1A, 2A and 3A).

### The group means of each dependent variable between young and older adults

The Table 2 showed the group means in each dependent variable particularly in condition 5 and 6 where the visual and somatosensory systems were perturbed simultaneously in two age groups: (1) the young group in our previous observations^14^ and (2) the older adults in the current study. The group means of the amount of sway variability in condition 5 and 6 were almost double in older adults than in young adults. In addition, the structure of sway variability was higher in young adults than in older adults in anterior-posterior direction. However, the structure of sway variability in medial-lateral direction showed no difference between young and older adults (Table 2).

### Interaction between MV and LSOT effect (Hypothesis #3)

We hypothesized that applying bilateral MV had larger impact than applying unilateral MV on older adults.

### Mean sway area

There was no significant interaction effect (Table 1).

### Amount and structure of sway variability

A significant interaction was identified between MV and LSOT (F = 4.22, *p* < 0.0001, Fig. 1C) in the amount of sway variability, in the structure of variability in the anterior-posterior direction (F = 36.05, *p* < 0.0001, Fig. 2C), but not in the structure of sway variability in the medial-lateral direction (Fig. 3C) We used Tukey post-hoc tests to perform the comparisons of no, unilateral, and bilateral MV effect in each condition. For the amount of sway variability, post-hoc comparisons showed that for normal unperturbed walking (LSOT Condition 1), MV did not produce any significant effect on the amount of sway variability. For all other LSOT conditions, both unilateral and bilateral MV significantly increased the amount of sway variability in comparison with no MV. In addition, for LSOT conditions 3, 4, and 5, bilateral and unilateral MV were not different from each other, while for LSOT conditions 2 and 6, bilateral MV produced a larger effect than the unilateral MV. For the structure of sway variability, all post-hoc comparisons were significant except the comparison between no MV and unilateral MV for LSOT condition 1. For the other five LSOT conditions, the unilateral MV produced significantly smaller values than the no MV condition. The bilateral MV produced significantly smaller values than both unilateral MV and control conditions for all LSOT conditions (Figs 1C, 2C and 3C).

## Discussion

We investigated how mastoid vibration (MV) could affect dynamic postural control in walking during simultaneous manipulation of the visual and the somatosensory systems in older adults. To accomplish this task we used three conditions of MV (none, unilateral, and bilateral) and combined them with our LSOT paradigm that systematically manipulates the visual and somatosensory systems^6^,^7^. We hypothesized that the MV would affect both the amount and temporal structure of sway variability during walking in older adults and, when applied in combination with manipulations of the visual and the somatosensory inputs, would produce similar but more pronounced observations as found in our previous work with young adults^14^. In addition, we expected that applying bilateral MV on older adults should have a stronger effect on dynamic postural control than applying unilateral MV.

Mean sway area was not affected by MV. However, a decrease in sway area was observed when both visual and somatosensory systems were simultaneously manipulated, but this was not statistically significant. The smallest mean sway area was observed in LSOT condition 5, where vision was reduced and somatosensory input manipulated at the same time. This result could be attributed to the fact that, mathematically, sway area highly depends on step length, which older adults have been found to decrease under reduced lighting conditions^15^.

Our hypotheses were partially supported. MV significantly increased the amount of sway variability in older adults. MV significantly affected the temporal structure of sway variability evident in a decrease in sample entropy (SampEn), however this was only observed in the AP direction. Decreased SampEn implies an increase in the rigidity of movement, and a reduction in the degrees of freedom of movement. MV augmented the effect of all conditions that involved visual and/or somatosensory manipulation, regardless of whether MV was presented unilaterally or bilaterally. The bilateral MV stimulation frequently produced larger effects than the unilateral. MV affected only measures of variability and not the mean sway area. In contrast to our previous observations in young adults^14^, the MV effect significantly decreased the SampEn values in older adults. This contrast indicates that younger and older adults may adopt opposing strategies in response to sensory manipulation. Specifically, older adults reduce their degrees of freedom, whereas younger adults increase them^14^. We also compared the preferred walking speed between the young adults from our previous study^14^ and the older adults from the present study to determine whether there was an a priori difference in the preferred walking speed between the two age groups. No significant differences were found (t = 1.587, p = 0.133; 1.02 ± 0.08 m/s. for young adults; 0.93 ± 0.09 m/s for older adults).

Our results showed that in all LSOT conditions MV increased the amount of sway variability during walking. In addition, the effect of MV was more pronounced in LSOT condition 2 and 5, both of which are associated with reduced vision. In our previous study, healthy young adults showed no significant increase in sway when standing with eyes closed in comparison to standing with eyes open^6^. However, the amount of sway variability during reduced and perturbed vision walking was significantly larger in comparison to normal walking^7^. Thus, the role of vision in standing postural control is not same as in locomotor postural control^7^,^16^,^17^. In healthy adults, vision plays crucial role to modulate the gait cycle, navigate the direction and avoid the obstacles^16^; however, for standing, the role of vision for keeping balance can be altered by other sensory systems^16^. Moreover, for patients with Acquired brain injury, better balance during standing is found with eye-closed in comparison with eye-opened^17^. However, these patients could not keep balance with eye-closed during walking^17^. Surprisingly, bilateral MV had a larger effect than unilateral MV only in LSOT conditions 2 (reduced vision) and 6 (vision and somatosensory are both manipulated). This is contrary to our findings in young adults^14^, for whom significantly larger effects were observed on application of MV in all LSOT conditions that involved sensory manipulations (i.e. all but Condition 1). We suspect that this result may be an effect of statistical power. From observation, there existed a trend such that bilateral MV affected the amount of sway variability in conditions where any sensory system was perturbed. Our results showed the power of MV and condition effect around 0.9 for the amount and temporal structure variability, but for sway area was around 0.9 for condition effect and 0.45 for MV effect. We believed we had decent power in this current study but might not exceed the level of significance of this particular situation. Of note, the amount of sway variability was considerably higher in this group, occasionally even doubling the values observed in our young adult population^14^, indicating that the older adults were much more challenged by our overall experimental design. These large increases in variability reflect a significant positional drift towards the front and the back of the treadmill; as sensory input is affected, positional information during locomotion is compromised. These results lead us to believe that MV, due to the affected vestibular input, causes confusion of the egocentric body-centered coordination system used during walking^18^,^19^. The larger increases in the amount of sway variability induced by MV in older adults may be due to a greater confusion of the egocentric body-centered coordinate system than that experienced by young adults^14^. This hypothesis is supported by Deshpande and Patla (2007) who demonstrated that vestibular input reweighting is less effective in older individuals in challenged walking than in young adults^4^.

Interestingly, we found that manipulation of the vestibular input through MV did not produce a significant effect on amount of sway variability unless combined with changes in another sensory input, reflected by our results in LSOT condition 1. This was also the case with the young adults of our previous study^14^. This suggests that MV in isolation might not pose a significant sensory input problem that manifests itself in this variable; at least not so big that other sensory systems could not compensate. Moreover, for older adults, we believe that the weighted sensory systems play a critical role in maintaining balance while receiving MV. The weighted vision may compensate the deficit of vestibular system to keep balance^20^.

Our results showed that MV decreased sample entropy values in the AP direction during walking. With bilateral MV this was the case for all LSOT conditions including condition 1. These changes in variability reflect significant alterations in the way positional drift towards the front and the back of the treadmill is temporally organized. Smaller values of sample entropy reflect more repeatability in the temporal structure with more regular net center of pressure (netCOP) trajectory patterns and increased movement rigidity. Importantly, this result opposes our findings in young adults^14^ in all LSOT conditions with the exception of condition 3. This suggests that the two age groups used different walking strategies to adjust their walking patterns while encountering sensory conflicts. Older adults tended to use conservative strategies and increase rigidity, whereas young adults tended to use exploratory strategies^8^. The relationship between optic flow and MV through visual and vestibular input interactions could explain the results for condition 3. It is known that manipulating optic flow affects the visual signal of self-motion^21^, which could evoke the well-known vection sensations of self-motion^22^ and after-rotation when walking^23^. When this is combined with MV, it may affect the egocentric body-centered coordinate system and further affect the pattern of natural locomotion^24^. However, in the present study the decreasing effect is uniform across all conditions and thus requires a more general explanation, which may be related to aging. Perception of the postural vertical, that provides an indicator measure of vestibular function in the absence of visual input and diminished somatosensory feedback, is affected in older adults^25^. There are also strong indications that aging results in deterioration of reciprocal cortical inhibition and decreases in the ability for multimodal vestibular integration of sensory inputs^26^. Thus, while applying MV on vestibular in older adults, the deteriorated sensory systems and sensory integrated capability forced older adults to select more conservative strategies, decrease degree of freedom in movement, to maintain dynamic balance.

Our results from both the amount and temporal structure of sway variability measures indicate that bilateral MV produces a larger effect than the unilateral. Research has shown that bilateral and unilateral MV can produce different locomotor outcomes^26^,^27^,^28^,^29^,^30^. In the context of our experimental design, the lesser effects observed with unilateral MV may be a result of the presence of external directional references provided by the set up (e.g. harness, corridor, orientation of the moving belt). These references could help the subject to readjust towards the AP direction^31^. This may also explain both the absence of a main effect of MV on the temporal structure of ML sway variability, and the small differences in the actual SampEn values between LSOT conditions in this direction. On the contrary, the bilateral MV, due to the production of a “pressing for forward” effect^29^, could produce much larger results since AP is the direction of motion.

The results for dynamic postural control in the AP direction were not replicated for the ML direction. Interestingly, the conditions that produced significant effects were those where the somatosensory system was perturbed (condition 4–6). This result indicates that the control mechanism between young and older adults may be different. For young adults, peripheral vision may play a dominant role in controlling locomotion to overcome the sensory restriction^32^,^33^. However, for older adult, both somatosensory and visual systems were both crucial to control posture in the ML direction. Specifically, older adults may have greater difficulty integrating the sensory information while both visual and somatosensory systems are simultaneously perturbed compared to young adults^14^. This corroborates similar findings in standing postural control research that older adults tend to make an extra step to maintain balance^12^ when both visual and somatosensory systems become unreliable, due to the deterioration of the ability to integrate sensory information^13^.

Finally, we would like to bring attention to the fact that the present results, when compared to our previous results with young adults^14^, produce contrasting findings. It has been suggested that a higher entropy value for older adults during standing may be interpreted as an effect of a more impaired sensory systems which provides less precise input for balance control^34^. The opposing result in this study, i.e. older adults showed lower entropy values, may similarly be due to a deterioration of sensory systems or their integration for balance control during walking. This contradictory result could be explained by the optimal movement variability theory^35^, that suggests that too much or too little variability may both be associated with impairment. Moreover, the MV effect on the temporal structure of variability was opposite for all LSOT conditions except LSOT condition 3 where MV produced a decreasing effect in both young and older adults. This is a very important finding as vestibular disorders have been difficult to diagnose, lacking a systematic assessment^36^, leading to the speculation that more than 1/3 of adults in the US aged 40 and older may be experiencing undiagnosed vestibular problems^37^. Our experimental design, and the results produced, could guide a more sensitive screening of vestibular system deterioration. Before such clinical translational efforts are made, however, the above conclusions should be tested by replication of our experiments with: over ground walking during which visual, somatosensory, and vestibular manipulations are introduced without the restrictions of the treadmill; galvanic vestibular stimulation^38^, dorsal neck muscles vibrations^39^,^40^, or changing head posture, known to affect balance and orientation responses^41^,^42^. These experiments will allow us to eliminate alternative hypotheses pertaining to the effect of the apparatus and the differences that exist between mastoid vibration and other stimulations to vestibular inputs.

## Methods

### Subjects

Ten healthy older adults (age 66.50 ± 4.32 years, height 1.72 ± 0.10 m and weight, 72.42 ± 20.93 kg) participated in this study. The average preferred walking speed (PWS) on a treadmill was 0.93 ± 0.09 m/s. Subjects were excluded from the study if they had a history of visual or vestibular deficits and scored above zero on the dizziness handicap inventory for a vestibular deficit^37^. This study was carried out in accordance with relevant guidelines and regulations of university of Nebraska medical center Institutional Review Board. In addition, all experimental protocols were approved by University of Nebraska medical center institutional review board. All subjects signed informed consent before experiments began.

### Instrumentation

The Locomotor Sensory Organization Test (LSOT) consists of two components: a virtual reality (VR) environment with a virtual corridor, and an instrumented treadmill (Bertec Corp., Columbus, OH, USA)^7^,^8^,^14^. The LSOT contains six conditions similar to the Sensory Organization Test to manipulate sensory information during walking:

1. Normal walking condition: both the speed of the virtual corridor and the treadmill speed are matched with the PWS.
2. Reduced visual condition: no VR is presented, the treadmill speed is matched with the PWS, and the subjects wear vision-reduced goggles. The vision was reduced by wearing a goggle (MSA Safety works, Pittsburgh, PA) with attaching two layers of windows film (Solutia Inc, St. Louis, MO). This design of goggle constrained the peripheral visual field, and reduced light intensity from 22 lux to 0.7 lux, measured using the Gossen Luna Pro light (Nurnberg, Germany).
3. Perturbed visual condition: achieved by manipulating the optic flow speed. The speed of the virtual corridor is pseudo-randomly varied between 80% and 120% of the selected PWS (restricted randomization between 80% and 120% in steps of 1%). Furthermore, these variations occur in pseudo-randomly assigned time intervals within 1 to 10 seconds (restricted randomization between 1 and 10 seconds in steps of 1 second^7^,^8^,^14^) in order to reduce likelihood of adaptation of walking in the perturbed environment. The treadmill speed is matched with the PWS.
4. Perturbed somatosensory condition: achieved by manipulating the treadmill speed. The speed of the virtual corridor is matched with the PWS. The treadmill speed is varied between 80% and 120% of the PWS in pseudo-randomly assigned time intervals within 1 to 10 seconds. This experimental design is justified as walking speed is highly associated with the sensitivity of the somatosensory system^43^.
5. Reduced visual and perturbed somatosensory condition: achieved by reducing vision and manipulating the treadmill speed. No VR is presented. The treadmill speed is varied between 80% and 120% of PWS in pseudo-randomly assigned time intervals within 1 to 10 seconds, and the subjects wear vision-reduced goggles.
6. Perturbed visual and somatosensory condition: achieved by manipulating optic flow and treadmill speed. Both the speed of the virtual corridor and the treadmill speed are varied between 80% and 120% of the selected PWS in pseudo-randomly assigned time intervals of 1 to 10 seconds. In this condition the velocity of the virtual corridor and treadmill are coupled with unity gain.

**Figure 4:**
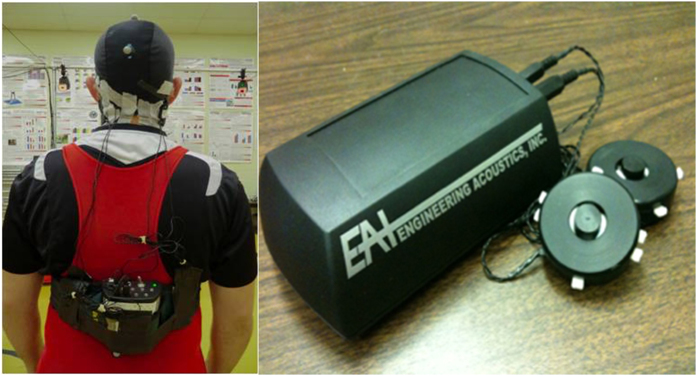
The tactor system contains two tactors and a controller unit for communication with the computer through Bluetooth and transmission of stimulus control signals to the tactors.

The MV used in the present study contained two electromechanical vibrotactile transducers (tactors; Engineering Acoustics, FL, USA.), that were placed on the mastoid processes bilaterally to perturb the vestibular feedback signals (Fig. 4). These tactors are designed for mounting within a seat or cushion, and can produce high force and displacement levels that allow the vibration to be easily felt even through layers of padding. The tactors require controllers and are designed for optimum vibrotactile efficiency at low frequencies (50–140 Hz). Their size is 4.8 cm in diameter and 1.9 cm in thickness. The frequency and amplitude of the stimulation are communicated wirelessly from a computer to the tactor controller unit, which transmits the signals through cables to the tactors. In the present study, the frequency and amplitude of MV were set to 100 Hz and 17.5 db respectively. This specific combination of frequency and magnitude was found in our pilot work and previous studies^14^,^44^,^45^ to be large enough to induce changes in eye movement and in postural control during standing. Firing was pulsed such that the duration of the firing and resting periods were 0.3 s and 0.6 s respectively in order to avoid saturating the sensation of the vestibular system. Three conditions of MV were applied: bilateral, unilateral or none (control). For unilateral stimulation, one side was randomly selected for each subject at the beginning of experiment and this side was consistent for all the unilateral trials.

Subjects wore a safety harness attached to a LiteGait system (Mobility Research, AZ, USA) to increase safety whilst on the treadmill.

### Procedures

Prior to data capture each subject walked for five minutes on the treadmill to determine their PWS. Subjects stood on the sides of the treadmill without touching the belts. Subsequently, the treadmill belt velocity was incremented from 0 to 0.8 m/s. Subjects were then asked to step onto treadmill while holding the handrail. After the subjects had started walking on the treadmill, they were asked to evaluate the speed using the following phrase: “Is this walking speed comfortable, like walking around the grocery store”? The treadmill velocity was then increased or decreased in 0.05 m/s increments following subject directions. Once a comfortable walking velocity had been attained, the subject walked continuously for 5 minutes.

Subjects were then required to complete trials under 18 randomly ordered conditions during the same visit. All subjects walked on the treadmill at their PWS for two minutes under each trial condition while data were captured. Between conditions, the subjects were asked to rest for one minute.

### Data Reduction

**Figure 5:**
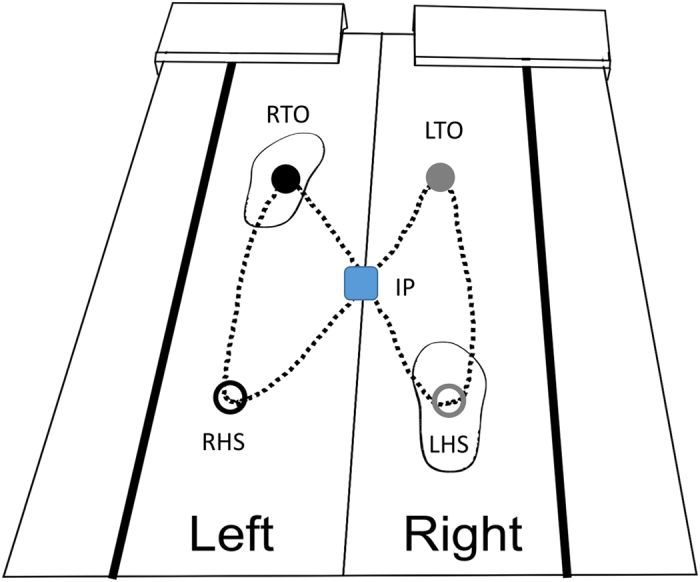
The netCOP sway area was composed from the two triangle areas that are represented by the dotted lines. Five points were used to generate these two-triangle areas as following: intersection point (IP), right heel-strike (RHS), right toe-off (RTO), left heel-strike (LHS), left toe-off (LTO).

The ground reaction force data acquired from the instrumented treadmill were low-pass filtered at 10 Hz with a 4th order Butterworth filter and used to estimate net center of pressure trajectories during each stride. The netCOP was defined as a point placement of the ground reaction vector. It represents a weighted average of pressures all over the force plate of the area in contact with the ground. netCOP sway area was calculated based on the motion of the center of pressure during the single limb support (SLS) phases of the stride^46^. The start and end positions of SLS on each limb were estimated using contralateral toe off events (LTO/RTO) and subsequent heel strike events (LHS/RHS). This resulted in four COP locations (LTO, LHS, RTO, RHS) for each stride, from which an intersection point (INT) was calculated. The netCOP sway area was determined by the area of two triangles bounded by LTO, LHS, INT and RTO, RHS, INT (Fig. 5). The mean and the standard deviation for each subject were calculated by averaging all available gait cycles. The netCOP sway variability was calculated as the coefficient of variation of netCOP sway area for each subject and was used as a metric of the amount of sway variability. In the current study, 85 gait cycles were used to calculate the netCOP sway variability. This was the lowest number of gait cycles performed by any participant in two minutes.

The temporal structure of sway variability was quantified using Sample Entropy (SampEn), calculated using a customized script in MatLab R2011a (Mathworks, Natick, MA). The position of the netCOP trajectory was divided into anterior-posterior and medial-lateral directions. SampEn was computed from the anterior-posterior netCOP trajectory and medial-lateral netCOP trajectory time series from the entire two minutes of available data for each trial. Data were downsampled from 12000 to 1200 data points as we had observed little physiological signal above 10 Hz during our pilot studies. In addition, there was no physiological signal above 10 Hz in the COP data in walking^14^,^34^. The SampEn algorithm is defined as the negative natural logarithm for conditional properties that a series of data points a certain distance apart, m, would repeat itself at m + 1^47^. Given the time series g(n) = g(1), g(2), …, g(N), where N is the total number of data points, a sequence of m-length vectors is formed. Vectors are considered alike if the tail and head of the vector are within the set tolerance level. The sum of the total number of like vectors is divided by m + 1 and defined as A or by N-m + 1 and defined as B. SampEn is then calculated as –ln(A/B). A time series with similar distances between data points would result in a lower SampEn value while large differences would result in higher SampEn values. There is no upper limit. A perfectly repeatable time series thus has a SampEn value equal to zero and a perfectly random time series has a SampEn value converging toward infinity. In the current study, a pattern length (m) of 2 and error tolerance of 0.2 were selected and used in the determination of SampEn values^47^.

### Statistical Analysis

Four two-way fully repeated measures ANOVAs (3 MV by 6 LSOT conditions/levels of analysis) were performed to determine statistical significance for the four dependent variables – mean netCOP sway area, coefficient of variation of the netCOP and the SampEn for the netCOP trajectory time series in the Anterior-Posterior and the Medial-Lateral directions. When significant main or interaction effects were determined, post-hoc comparisons were performed using the Tukey method. Statistical analysis was completed in SPSS 18.0 (IBM Corporation, Armond, NY). The Shapiro-Wilk Normality Test was used to test the normality of each dependent variable, with the alpha value set at 0.05.

## Additional Information

**How to cite this article**: Chien, J. H. *et al*. Mastoid vibration affects dynamic postural control during gait in healthy older adults. *Sci. Rep.* **7**, 41547; doi: 10.1038/srep41547 (2017).

**Publisher's note:** Springer Nature remains neutral with regard to jurisdictional claims in published maps and institutional affiliations.

## Figures and Tables

**Table 1:** Group condition means for netCOP sway area for 85 gait cycles per subject (m^2^).

Conditions	LSOT 1#	LSOT 2	LSOT 3	LSOT 4	LSOT 5!	LSOT 6
	Normal Walking	Reduced Vision	Perturbed Vision	Perturbed somatosensation	Reduced vision, perturbed somatosensation	Perturbed vision, perturbed somatosensation
No MV	0.0444 ± 0.006	0.0434 ± 0.005	0.0451 ± 0.004	0.0445 ± 0.005	0.0416 ± 0.006	0.0427 ± 0.004
Unilateral MV	0.0459 ± 0.005	0.0417 ± 0.003	0.0436 ± 0.005	0.0436 ± 0.004	0.0399 ± 0.005	0.0410 ± 0.005
Bilateral MV	0.0443 ± 0.005	0.0412 ± 0.005	0.0423 ± 0.005	0.0430 ± 0.005	0.0400 ± 0.005	0.0406 ± 0.005

A significant main effect was found only for LSOT condition. No interaction effect was present. Post-hoc analysis using pairwise Tukey comparisons revealed significant differences between conditions LSOT 1 and LSOT 5.

LSOT: Locomotor Sensory Organization Test; MV: Mastoid Vibration.

^!^Significant difference exhibited when compared to LSOT condition 1.

^#^Significant difference exhibited when compared to LSOT condition 5.

**Table 2:** Group means of each dependent variable in young and older adults in conditions where visual and somatosensory systems were perturbed simultaneously.

	Amount of sway variability		Structure of sway variability in anterior-posterior direction		Structure of sway variability in medial-lateral direction	
	Condition 5	Condition 6	Condition 5	Condition 6	Condition 5	Condition 6
Young*	17.07 ± 2.52	15.91 ± 1.60	0.37 ± 0.03	0.36 ± 0.02	0.07 ± 0.01	0.06 ± 0.01
Older	30.08 ± 3.71	26.39 ± 2.81	0.32 ± 0.02	0.29 ± 0.01	0.07 ± 0.01	0.06 ± 0.01

Condition 5: walking with reduced vision and perturbed somatosensation.

Condition 6: walking with perturbed vision and perturbed somatosensation.

^*^The young data is from our previous observation^14^.
